# Construction and assessment of an angiogenesis-related gene signature for prognosis of head and neck squamous cell carcinoma

**DOI:** 10.1007/s12672-024-01084-z

**Published:** 2024-07-16

**Authors:** Kaiqin Wang, Ruizhe Zhang, Changya Li, Huarong Chen, Jiafeng Lu, Houyu Zhao, Xianlu Zhuo

**Affiliations:** 1 Department of Otolaryngology, The Affiliated Hospital of Guizhou Medical University, Guiyang, Guizhou China; 2 Department of Otolaryngology, Anshun People’s Hospital, Anshun, Guizhou China

**Keywords:** Head and neck squamous cell carcinoma (HNSC), Angiogenesis, Prognosis, Treatment, Drug resistance, Tumor microenvironment (TME)

## Abstract

**Objective:**

Angiogenesis-associated genes (AAGs) play a critical role in cancer patient survival. However, there are insufficient reports on the prognostic value of AAGs in head and neck squamous cell carcinoma (HNSC). Therefore, this study aimed to investigate the correlation between AAG expression levels and survival in HNSC patients, explore the predictive value of signature genes and lay the groundwork for future in-depth research.

**Methods:**

Relevant data for HNSC were obtained from the databases. AAGs-associated signature genes linked to prognosis were screened to construct a predictive model. Further analysis was conducted to determine the functional correlation of the signature genes.

**Results:**

The signature genes (STC1, SERPINA5, APP, OLR1, and PDGFA) were used to construct prognostic models. Patients were divided into high-risk and low-risk groups based on the calculated risk scores. Survival analysis showed that patients in the high-risk group had a significantly lower overall survival than those in the low-risk group (P < 0.05). Therefore, this prognostic model was an independent prognostic factor for predicting HNSC. In addition, patients in the low-risk group were more sensitive to multiple anti-cancer drugs. Functional correlation analysis showed a good correlation between the characteristic genes and HNSC metastasis, invasion, and angiogenesis.

**Conclusion:**

This study established a new prognostic model for AAGs and may guide the selection of therapeutic agents for HNSC. These genes have important functions in the tumor microenvironment; it also provides a valuable resource for the future clinical trials investigating the relationship between HNSC and AAGs.

## Introduction

Head and neck squamous cell carcinoma (HNSC) is the most prevalent type of cancer in the head and neck region (mouth, lips, nose, throat and larynx) [1]. Recent studies reveal a rising prevalence of HNSC, resulting in morbidity and mortality rates ranked among the top 10 worldwide malignancies [2]. The extremely grave nature of HNSC, coupled with inadequate presence of initial symptoms, often leads to a delayed diagnosis. Although HNSC patients have a better prognosis following surgical and radiotherapy treatment, their 5 year survival rate remains below 50% [3]. The cancer cells in HNSC can metastasize through the lymphatic and blood systems, and metastasis is one of the main factors for poor patient prognosis. It is worth noting that tumor metastasis is closely related to angiogenesis. Angiogenesis provides a pathway for tumor metastasis, and metastasis promotes angiogenesis [4].

Angiogenesis refers to the formation of new blood vessels from existing capillaries or post-capillary venules [5]. Tumor growth, invasion, and metastasis necessitate a blood supply containing an adequate amount of oxygen and nutrients. Henceforth, neovascularization is crucial for the continuous survival and development of tumor cells [6]. The survival of gastric cancer patients has been shown to be affected by angiogenesis-associated genes (AAGs) [7]. Angiogenesis is not only associated with gastric cancer, but also with the progression of a variety of tumors, including non-small cell lung cancer (NSCLC), breast cancer, renal cell carcinoma and other tumors. Angiogenesis plays a critical role in the growth and metastasis of NSCLC, and increased angiogenesis suggests that patients have a poor prognosis [8]. In breast cancer, tumor proliferation and metastasis are inhibited by suppressing the expression of AAGs [9]. The expression of AAGs is increased in renal cell carcinoma and has an impact on patient prognosis [10]. Additionally, angiogenesis inhibitor therapy can enhance the survival rate of cancer patients, including those with colorectal and liver cancer [11]. The combination of anti-angiogenesis therapy with conventional radiotherapy was evidenced to improve outcomes for cancer patients [12].

There are several AAGs involved in the process of angiogenesis, and Kim S et al. discovered that Versican (VCAN) enhances tumor cell growth, angiogenesis, and promotes tumor metastasis [13]. VCAN is a highly expressed gene in cancers such as bladder and ovarian cancer, and high VCAN expression can lead to poor patient prognosis [14, 15]. The pro-angiogenic factor vascular endothelial growth factor A (VEGFA) promotes the proliferation of vascular endothelial cells and has been implicated in the progression and metastasis of various cancers. Elevated VEGFA expression is linked to unfavorable patient prognosis [16]. Aberrant expression of AAGs is involved in tumor angiogenesis and affects patient prognosis. Therefore, it is crucial to identify the AAGs that affect the prognosis of HNSC.

Currently, the prognostic value of AAGs in HNSC has rarely been reported. Thus, studying AAGs is of great significance. The aim of this study was to investigate the relationship between the expression levels of AAGs and the survival of HNSC patients and to explore the prognostic value of the characterized genes.

## Materials and methods

### Data collection and collation

Transcriptomic data FPKM (RNA-Seq) was obtained from 566 cases of HNSC and para-cancer tissues downloaded from The Cancer Genome Atlas (TCGA) database (https://portal.gdc.cancer.gov), as well as associated clinical data for 528 cases. The HNSC dataset GSE41613 and its associated platform files were obtained from the Gene Expression Omnibus (GEO) database (https://www.ncbi.nlm.nih.gov/geo/). GSE41613 is an expression profiling dataset from the FHCRC, which includes 97 patients with high-risk HPV-negative OSCC. There were 66 males and 31 females out of the 97 patients, and their ages ranged from 19 to 88 years old, while the follow-up time was 13 days to 7 years. 41 patients were stage I/stage II, 11 and 56 were stage III/stage IV. Following the exclusion of seven cases with unknown causes of death and 14 cases with non-oral cancer deaths, the validation set was reduced to 76 cases [17]. Additionally, a literature review identified thirty-six AAGs [7].

### Construction of a prognostic model for AAGs

Initially, RNA-Seq expression profiles of 36 AAGs in both tumor and non-tumor samples were obtained from the training set. Subsequently, angiogenesis-related differentially expressed genes (DEGs) were screened, and from this list, genes with prognostic value were identified. The study employed LASSO regression analysis to identify the most promising trait genes for prognosis and to develop prognostic models based on their regression coefficients.

### Validation of the model

We determined the risk score for each patient with HNSC using the formula: risk score = esum (expression of each gene × corresponding coefficient). Patients from both the training and validation sets were classified into high and low risk groups based on the median risk score of the training set. To assess the effectiveness of the feature genes in discriminating between patients in the high and low-risk groups, we conducted Principal Component Analysis (PCA) and t-SNE analyses. Subsequently, we evaluated the prognostic ability of the model using the Kaplan–Meier survival curve and time-dependent receiver operating characteristic (ROC) analysis. The study utilized the Kaplan–Meier survival curve to evaluate any disparities in overall survival (OS) between the high-risk and low-risk groups. Additionally, we employed the ROC to anticipate patient survival rates at 1, 3, and 5 years by computing the area under curve (AUC). Finally, a nomogram was constructed grounded on the prognostic model and clinicopathological features to anticipate the likelihood of patient survival less than 1, 3, and 5 years. Decision curve analysis (DCA) and C-index values were also employed to appraise the precision of the nomogram.

### Gene set enrichment analysis (GSEA)

GSEA was conducted using the R package to investigate the signaling pathways in the high and low-risk groups. We identified the top five pathways relevant to tumor development and progression.

### Immunological analysis

We employed single-sample gene-set enrichment analysis (ssGSEA) to compute scores for immune cells and pathways associated with immune function. Immune differential analysis was conducted to detect variations in immune cells and pathways related to immunity within high and low risk groups. In addition, we validated the immune cell differential analysis using the EPIC algorithm in Sangerbox (http://sangerbox.com/) [18].

### Drug sensitivity analysis

Furthermore, we performed a drug sensitivity analysis to evaluate the effectiveness of frequently used chemotherapeutic agents for treating HNSC in individuals with high and low risk. To calculate the maximal half-inhibitory concentration (IC_50_) values of these agents, we deployed the R package ‘‘pRRophetic’’.

### Functional analysis of characteristic genes

We first evaluated the protein expression of the signature genes. Later, we examined the connections among the signature genes and metastasis, invasion, and angiogenesis in HNSC. Next, we explored the possible correlation between signature genes and immune cells. Furthermore, we analyzed the association between signature genes and CD34, fibronectin 1 (FN1), lysyl oxidase-like protein 2 (LOXL2), and VCAN in HNSC. Finally, a comparison was made between the expression of the signature genes in normal, HNSC, and metastatic tissues.

### Single-cell analysis

Tumor Immune Single-cell Hub 2 (TISCH2, http://tisch.comp-genomics.org/) was employed to facilitate tumor microenvironment (TME) single-cell transcriptome analysis [19]. In this research, we analyzed the expression of five identified genes in the HNSC TME by utilizing the TISCH2 database.

### Statistical analysis

The data were processed using the Perl software (version 5.30.0). R software (version 4.2.0) and related packages were used for statistical analyses. Statistical significance was conventionally established at P < 0.05.

## Results

### Identification of prognosis-related AAGs

**Fig. 1:**
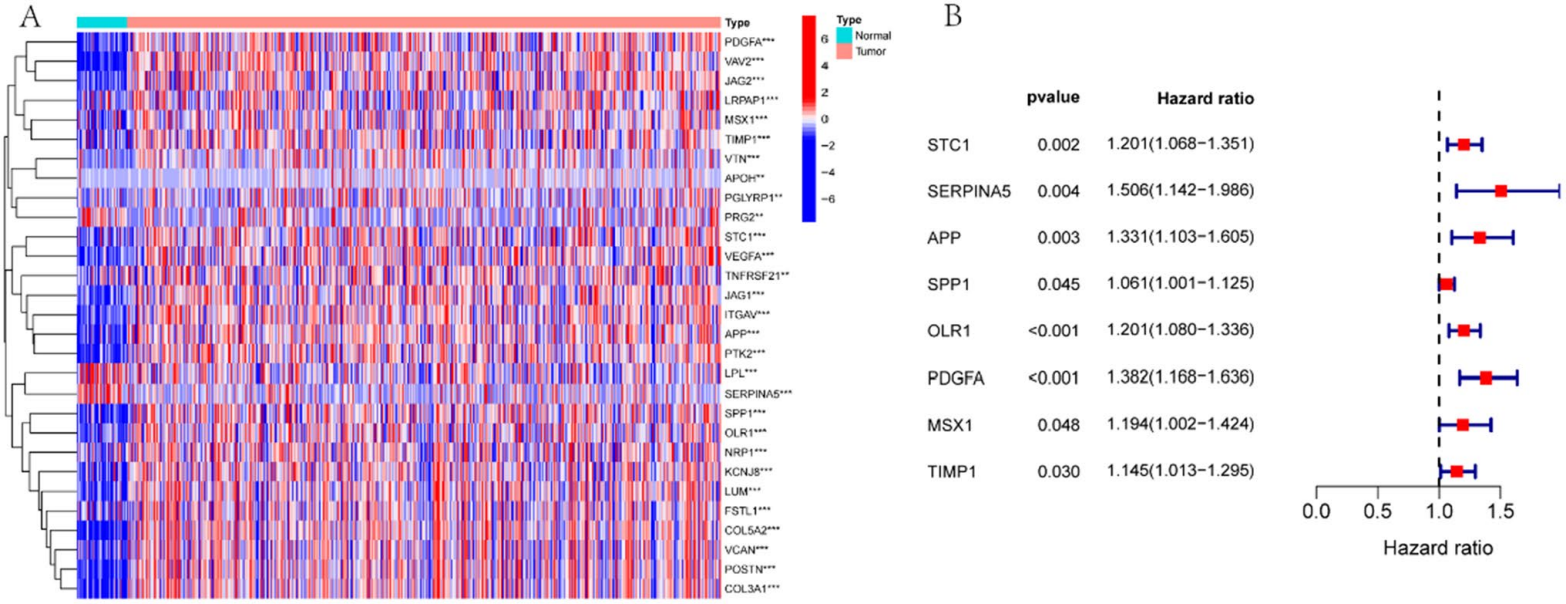
Analysis based on the TCGA dataset (training set). **A**. Heat map of differences between DEGs in non-tumor samples (represented by N, indicated by blue) and tumor samples (represented by T, indicated by red). (* represents p < 0.05, ** represents p < 0.01, *** represents p < 0.001). **B**. Prognosis-related AAGs

Initially, we isolated the expression of AAGs in both tumor and non-tumor samples from the training set. Subsequently, we assessed the differences in AAGs, identifying a total of 29 DEGs (P < 0. 05). Among these genes, 24 were upregulated, which included VCAN, POSTN, FSTL1, LRPAP1, STC1, VEGFA, TNFRSF21, COL5A2, ITGAV, KCNJ8, APP, JAG1, COL3A1, SPP1, NRP1, OLR1, PDGFA, PTK2, PGLYRP1, VAV2, MSX1, TIMP1, JAG2, and LUM. Furthermore, five DEGs were down-regulated (including PRG2, LPL, APOH, VTN, and SERPINA5; Fig. 1A). Next, ten genes linked to prognosis were examined, namely APP, OLR1, STC1, PDGFA, SERPINA5, SPP1, S100A4, MSX1, TIMP1, and APOH (Fig. 1B). The intersection of DEGs with prognosis-related genes led to the identification of eight prognosis-related DEGs, namely STC1, SERPINA5, APP, SPP1, OLR1, PDGFA, MSX1, and TIMP1.

### Construction and validation of prediction models

**Fig. 2:**
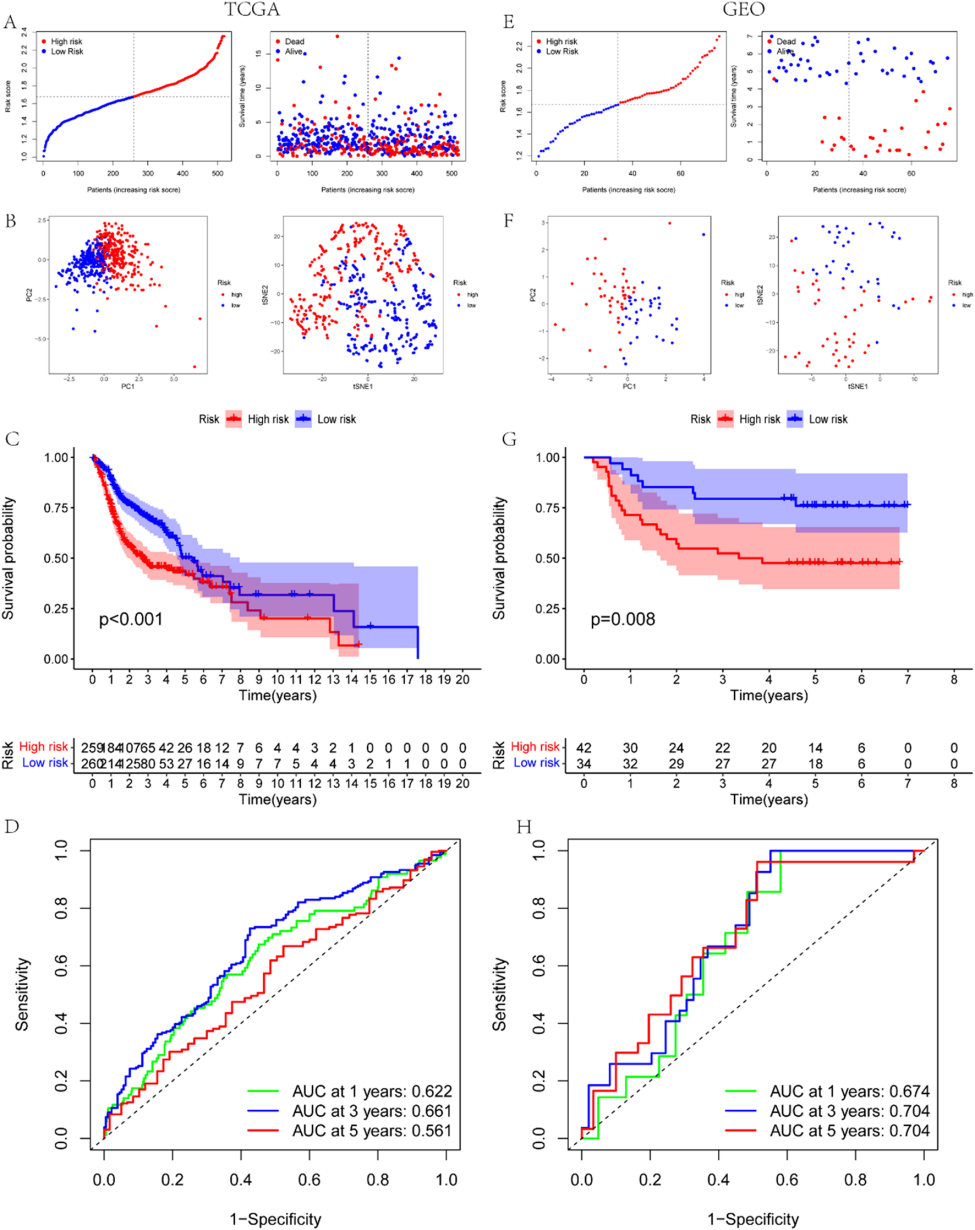
Survival analysis based on the TCGA dataset, and external validation of prognostic models based on the GEO dataset: **A**, **E**. Distribution of HNSC patients and patient survival based on risk scores (low-risk group: on the left of the dashed line; high-risk group: on the right of the dashed line). **B**, **F**. PCA and t-SNE analysis. **C**, **G**. Comparison of Kaplan–Meier survival curves for OS in HNSC between high and low-risk groups. **D**, **H**. Time-dependent ROC curves for HNSC patients

These 8 prognostic genes play an important role in the OS of HNSC patients. Firstly, LASSO Cox regression analysis was performed on these genes. The optimal λ value was established, comprising of five genes, namely STC1, SERPINA5, APP, OLR1, and PDGFA. These five genes were utilized to devise a risk assessment model for AAGs. The risk score was then calculated using this formula: risk score = (0.09976 * APP exp.) + (0.09231 * OLR1 exp.) + (0.18165 * PDGFA exp.) + (0.03183 * STC1exp.) + (0.14590 * SERPINA5 exp.). Patients in both the training (Sourced from the TCGA database) and validation (Sourced from the GEO database) sets were classified into high-risk and low-risk groups based on the median risk score of the training set. The number of patients who died increased as the risk score increased (Fig. 2A, E). Our evaluation of PCA and t-SNE exhibited that the genes utilized to form the model were successful in distinguishing two clusters of HNSC patients: those at high risk and those at low risk (Fig. 2B, F). The Kaplan–Meier analysis confirmed that patients in the low-risk group had a significantly longer OS than those in the high-risk group (P < 0.05, Fig. 2C, G). The predictive ability of the model was assessed using the ROC curve and the AUC was 0.633, 0.691, and 0.581 at 1, 3, and 5 years, respectively (Fig. 2D, H).

### Prognostic value of the model

**Fig. 3:**
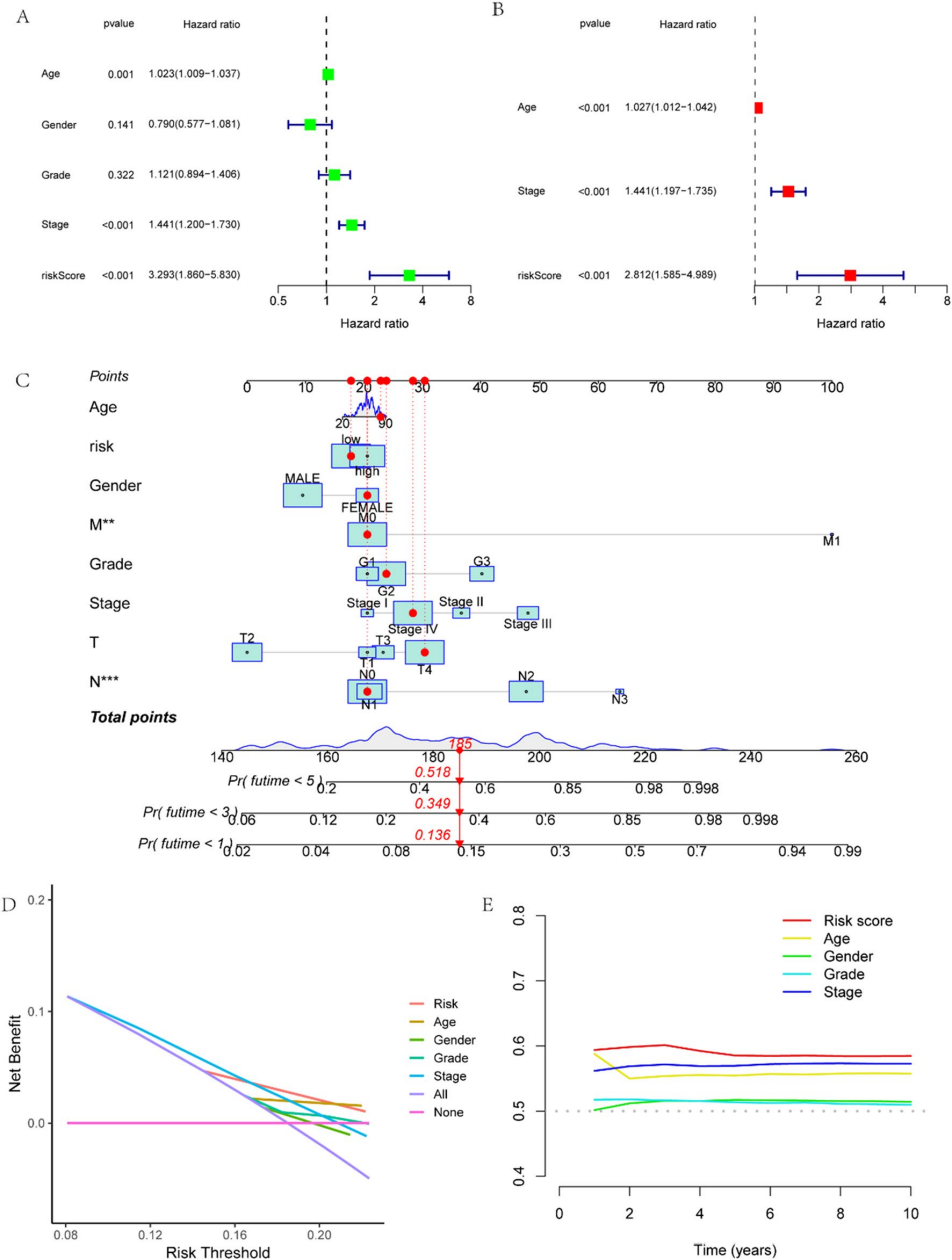
**A**. Univariate cox regression analysis. **B**. Multivariate cox regression analysis (a and b are univariate and multivariate cox regression analyses of risk scores based on the TCGA dataset). **C**. Nomogram constructed from risk scores and associated clinicopathological features (For this patient, with a total score of 185, the probability of survival at less than 1, 3, and 5 years was 13.6%, 34.9%, and 51.8% respectively). **D**. DCA of the nomogram. **E**. C-index values of the nomogram

Univariate and multivariate Cox regression analyses were used to determine whether prognostic models could be an independent prognostic factor for patients with HNSC. In the training dataset, the univariate Cox regression analysis revealed that age (HR = 1.023, 95% CI 1.009–1.037), tumor pathological stage (HR = 1.441, 95% CI 1.200–1.730), and risk score (HR = 3.293, 95% CI 1.860–5.830) could be potential prognostic indicators for HNSC patients (Fig. 3A). Multivariate Cox regression analysis revealed that age (HR = 1.027, 95% CI 1.013–1.042), tumor pathological stage (HR = 1.441, 95% CI 1.197–1.735), and risk score (HR = 2.812, 95% CI 1.585–4.989) were independent prognostic factors for HNSC patients (Fig. 3B). Based on the above analysis, the prognostic model can function as a stand-alone predictor for HNSC patients. A nomogram was constructed based on age, gender, tumor pathological stage, grade, T stage, N stage, M stage, and risk score of the patients. The results show that the nomogram can accurately predict the OS of patients at less than 1, 3, and 5 years (Fig. 3C). The DCA and C-index values were generated by merging relevant clinicopathological data (Fig. 3D, E). The DCA and C-index plots demonstrate that our constructed model for predicting patient survival outperforms other clinicopathological information.

### Association between functional annotation of signature genes and immune cell infiltration

**Fig. 4:**
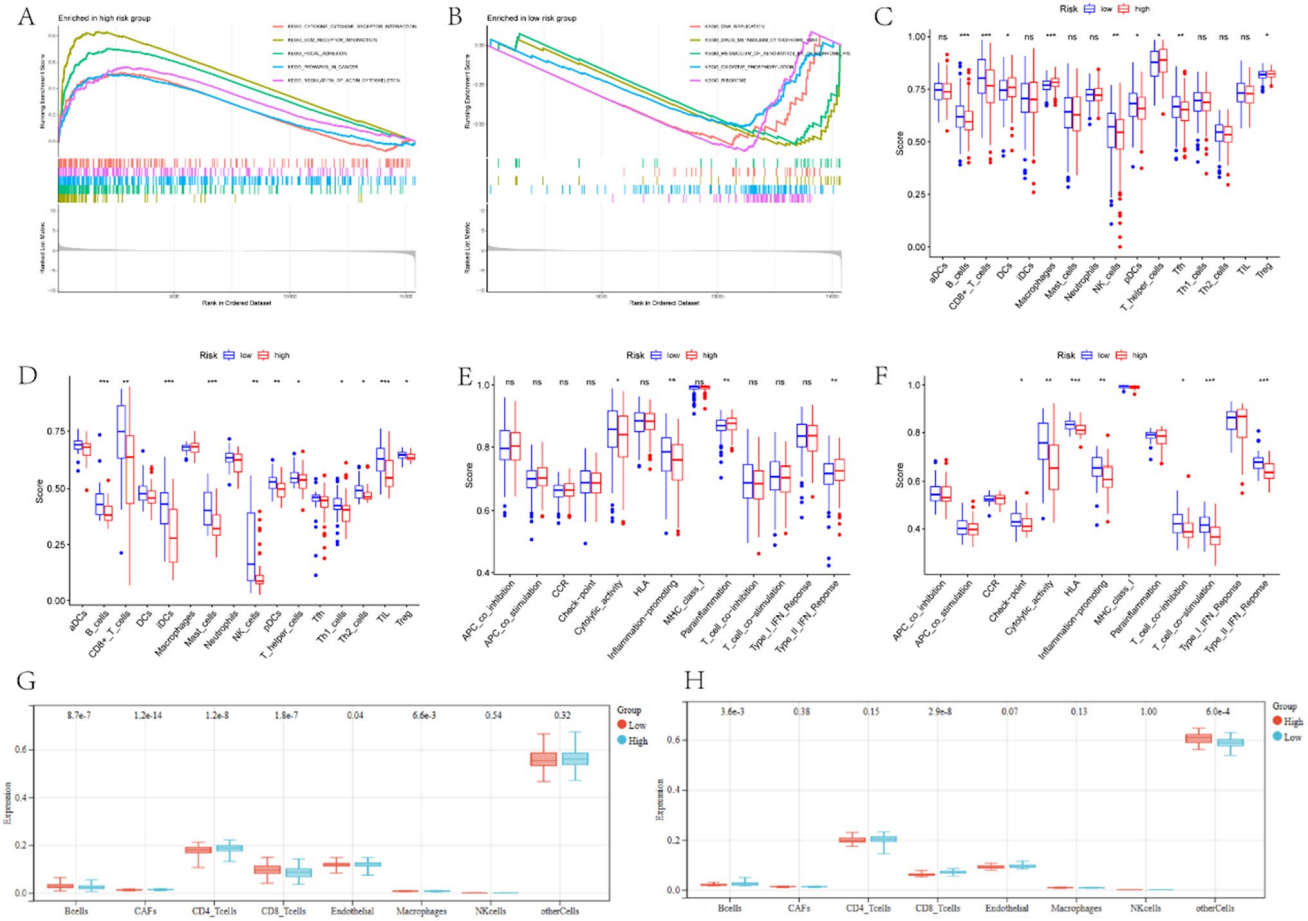
**A**. Top 5 pathways enriched in high-risk group. **B**. Top 5 pathways enriched in low-risk group. Comparison of the enrichment scores of immune cells in the training set **C**, **G** and validation set **D**, **H** for the low-risk groups and the high-risk groups. Comparison of immune function enrichment scores between low-risk and high-risk groups in training set **E** and validation set **F**. (* represents p < 0.05; ** represents p < 0.01; *** represents p < 0.001)

The GSEA analysis shows that the high-risk group is mainly involved in the cytokine-cytokine-receptor-interaction, ECM-receptor-interaction, focal-adhesion, pathways-in-cancer, and regulation-of-actin-cytoskeleton pathways (Fig. 4A); whereas the low-risk group was mainly involved in the Drug-metabolism-cytochrome-P450, metabolism-of-xenobiotics-by-cytochrome-P45, oxidative-phosphorylation, DNA-replication, and ribosome access pathways (Fig. 4B).

We performed ssGSEA and immune difference analysis on both the training and validation datasets. The results indicate that the high-risk group had significantly lower levels of immune infiltration of B cells, CD8 + T cells, NK cells, and Treg than the low-risk group (Fig. 4C, D). The EPIC algorithm also showed that the immune infiltration levels of B cells and CD8 T cells were significantly lower in the high-risk group than in the low-risk group (Fig. 4G, H). Additionally, cytolytic activity and pro-inflammatory activity were significantly lower in the high-risk group than in the low-risk group (Fig. 4E, F). Thus, the aforementioned findings display a relationship between the level of HNSC and several immune cell infiltrations in the cohort with a high risk. Additionally, the data imply that the infiltration of these immune cells could potentially be linked to tumor angiogenesis.

### Drug sensitivity analysis

**Fig. 5:**
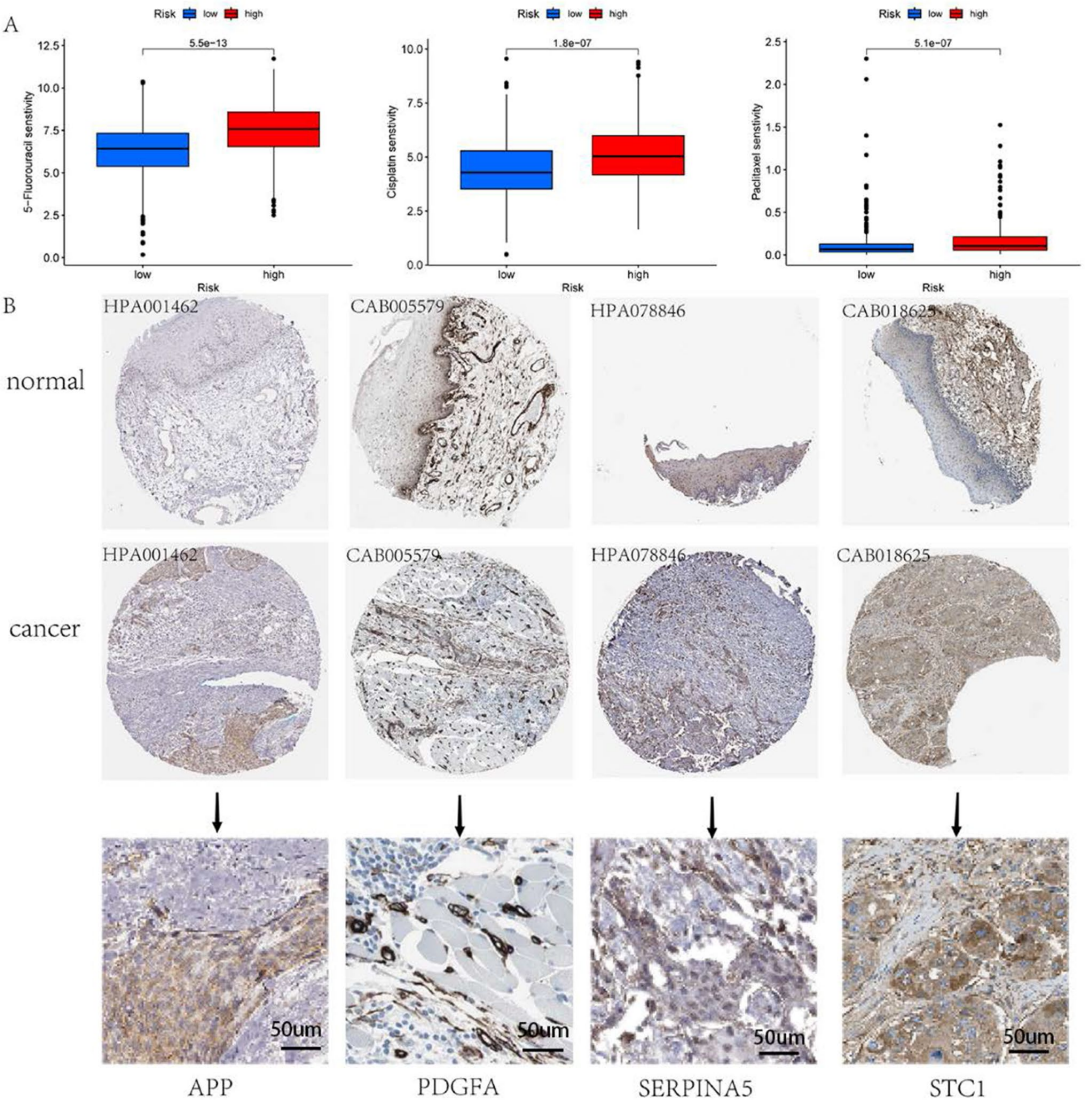
**A**. 5-Fluorouracil, Cisplatin and Paclitaxel in different risk groups. **B**. The protein levels of APP, PDGFA, SERPINA5, and STC1 in HNSC tissue in HPA

To evaluate the efficacy of commonly used chemotherapeutic drugs for the treatment of HNSC in high and low risk populations, our analysis implemented the ‘‘pRRophetic’’ R-package. The results of the study indicate that patients in the low-risk category exhibited an elevated sensitivity to chemotherapy comprising 5-Fluorouracil, Cisplatin, and Paclitaxel in comparison to those in the high-risk category (Fig. 5A).

### Functional analysis of signature genes

The protein expression of the characterized genes was assessed using immunohistochemistry in the Human Protein Atlas (HPA, https://www.proteinatlas.org) tool [20]. According to the database, the immunohistochemical staining data were based on the proteins APP (normal: Staining is low, Quantity < 25%; cancer: Staining is medium, Quantity 25–75%), PDGFA (normal: Staining is medium, Quantity 25–75%; cancer: Staining is high, Quantity > 75%), SERPINA5 (normal: Staining is not detected, Quantity < 25%; cancer: Staining is low, Quantity 25–75%), and STC1 (normal: Staining is low, Quantity < 25%; cancer: Staining is medium, Quantity 25–75%). Staining intensity was found to be higher in HNSC tissues than in normal tissues based on intensity and percentage of staining using semi-quantitative methods. These genes were found to be located in the cytoplasmic/membrane region of cancer cells. There are currently no relevant protein expression data for OLR1 in the HPA database (Fig. 5B).

**Fig. 6:**
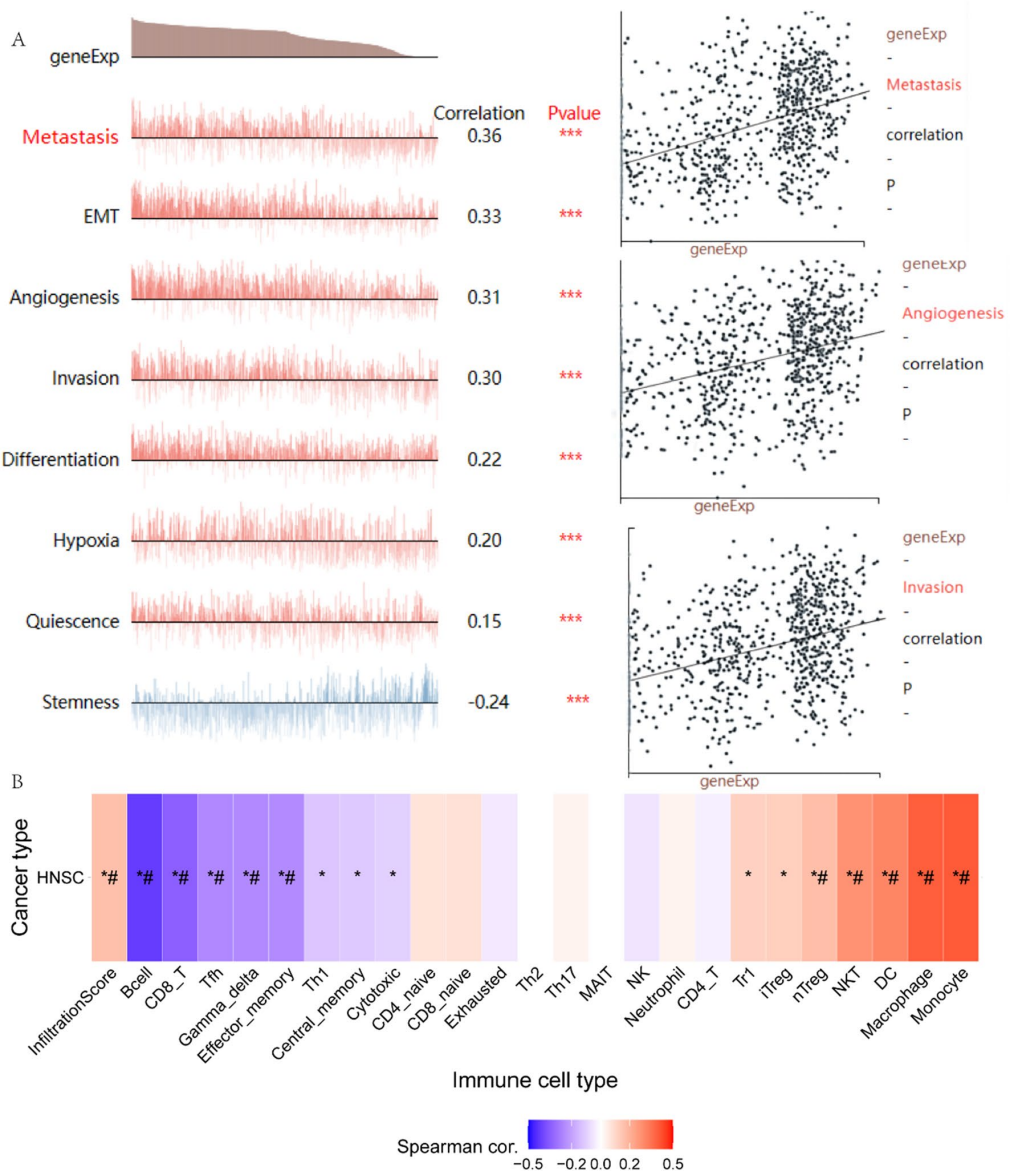
**A**. Functional status of characterized genes (APP, PDGFA, OLR1, STC1, and SERPINA5) in HNSC analyzed based on CancerSEA database. **B**. Exploring the correlation between immune infiltration and GSVA scores of gene sets (APP, PDGFA, STC1, OLR1, and SERPINA5) based on the GSCA database (‘‘#’’ represents FDR ≤ 0.05)

Secondly, we performed functional analyses on known genes to investigate the association of these genes with metastasis, invasion, and angiogenesis in HNSC. We investigated the correlations between characterized genes and invasion, metastasis, and angiogenesis in HNSC by searching the CancerSEA database (http://biocc.hrbmu.edu.cn/CancerSEA/) [21]. The results showed correlation coefficients of 0.36, 0.30, and 0.31 for APP, PDGFA, STC1, OLR1, and SERPINA5 with respect to metastasis, invasion, and angiogenesis (P < 0.05). These genes were consequently found to positively correlate with metastasis, invasion, and angiogenesis in HNSC patients (Fig. 6A).

Next, we used Gene Set Cancer Analysis (GSCA, http://bioinfo.life.hust.edu.cn/GSCA/#/.) to investigate the relationship between immune infiltration and gene set (defined genes APP, PDGFA, STC1, OLR1, and SERPINA5) expression scores (GSVA scores) [22]. In our investigation of HNSC, we observed a significant positive correlation between the GSVA enrichment score and the abundance of macrophages and monocytes. In contrast, CD8-T and B cells exhibited a significant negative correlation with the GSVA enrichment score (absolute value of correlation coefficient greater than or equal to 0.1, P < 0.05; Fig. 6B).

**Fig. 7:**
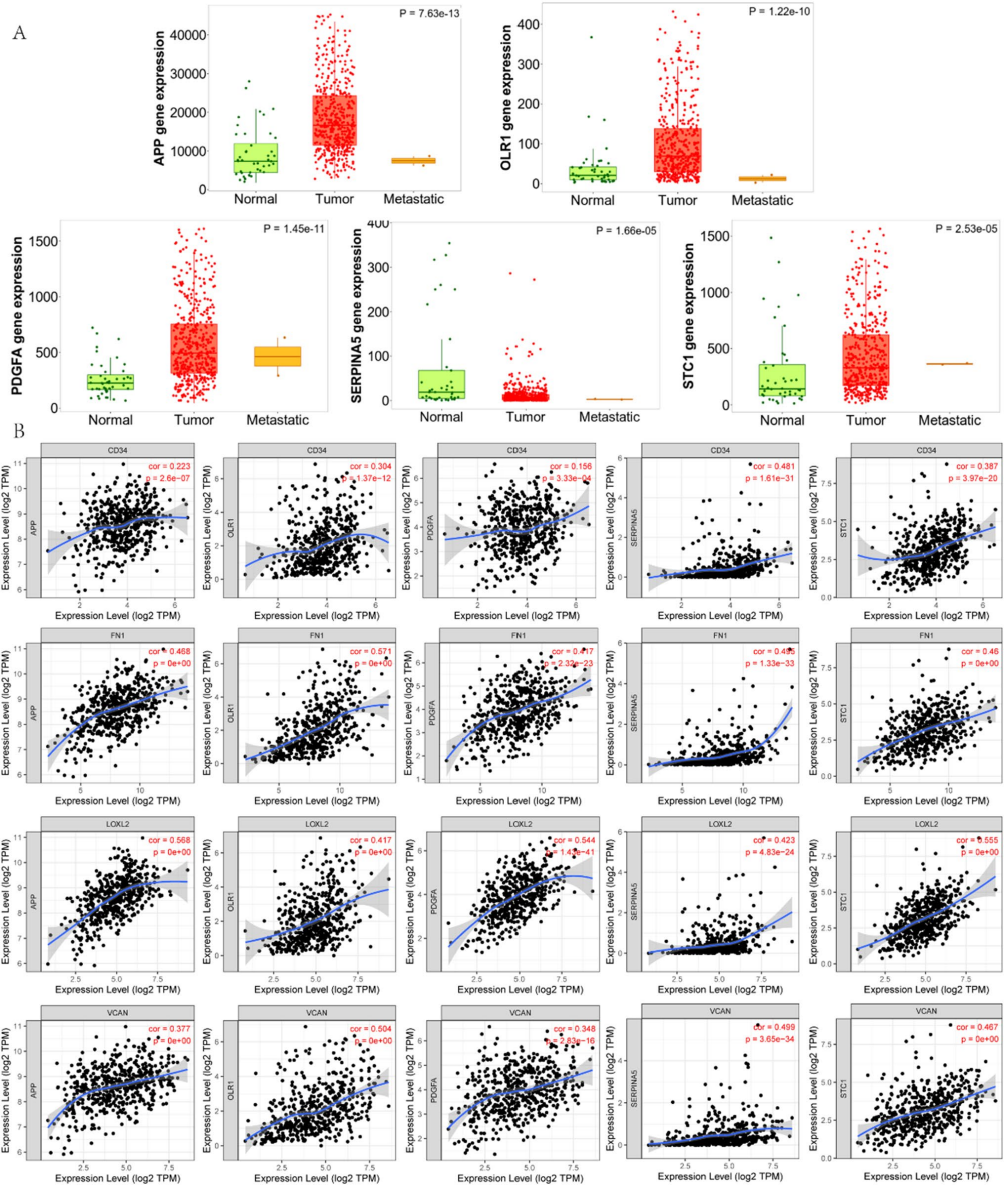
**A**. Expression of APP, OLR1, PDGFA, SERPINA5, and STC1 in normal, HNSC, and metastatic tissue. **B**. Correlation of APP, OLR1, PDGFA, SERPINA5, and STC1 with CD34, FN1, LOXL2, and VCAN in HNSC

Again, RNA-seq data from HNSC were analyzed using the Tumor, Normal, and Metastatic tissues (TNMplot) database (https://tnmplot.com/analysis/) to investigate the expression of the characterized genes in normal, HNSC, and metastatic tissues in a factual and concise manner [23]. The study showed that the genes APP, PDGFA, OLR1, STC1, and SERPINA5 were upregulated in HNSC cases. The genes APP (P = 7.63e−13), OLR1 (P = 1.22e−10), PDGFA (P = 1.45e−11), SERPINA5 (P = 1.66e−05), and STC1 (P = 2.53e−05) showed differential expression in normal, HNSC and metastatic tissues (P < 0.05). Two-way analysis revealed differential expression of five signature genes in both normal and HNSC tissues. Additionally, SERPINA5 had differential expression in normal and metastatic tissues, while APP as well as OLR1 had differential expression in tumor and metastatic tissues (P < 0.05, Fig. 7A).

Finally, the Tumor Immune Estimation Resource (TIMER) database (http://timer.cistrome.org/) was used to investigate the association of the characterized genes with CD34, FN1, LOXL2, and VCAN in HNSC [24]. The results show a positive correlation between CD34 and APP (r = 0.223, P = 2.6e−07), OLR1 (r = 0.304, P = 1.37e-12), PDGFA (r = 0.156, P = 3.33e−04), SERPINA5 (r = 0.481, P = 1.61e-31), and STC1 (r = 0.387, P = 3.97e−20) in HNSC tissues. APP (r = 0.468, P = 0e + 00), OLR1 (r = 0.571, P = 0e + 00), PDGFA (r = 0.417, P = 2.32e−23), SERPINA5 (r = 0.495, P = 1.33e−33), and STC1 (r = 0.46, P = 0e + 00) and FN1 showed a positive correlation in HNSC tissues. Additionally, APP (r = 0.568, P = 0e + 00), OLR1 (r = 0.417, P = 0e + 00), PDGFA (r = 0.544, P = 1.43e−41), SERPINA5 (r = 0.423, P = 4.83e−24), and STC1 (r = 0.555, P = 0e + 00), and LOXL2 also exhibited a positive correlation in HNSC tissue. APP (r = 0.377, P = 0e + 00), OLR1 (r = 0.504, P = 0e + 00), PDGFA (r = 0.348, P = 2.83e−16), SERPINA5 (r = 0.499, P = 3.65e−34), and STC1 (r = 0.467, P = 0e + 00) demonstrated a favorable association with VCAN in HNSC tissue samples (Fig. 7B).

### Correlation of five characterized genes with the HNSC tumor microenvironment

**Fig. 8:**
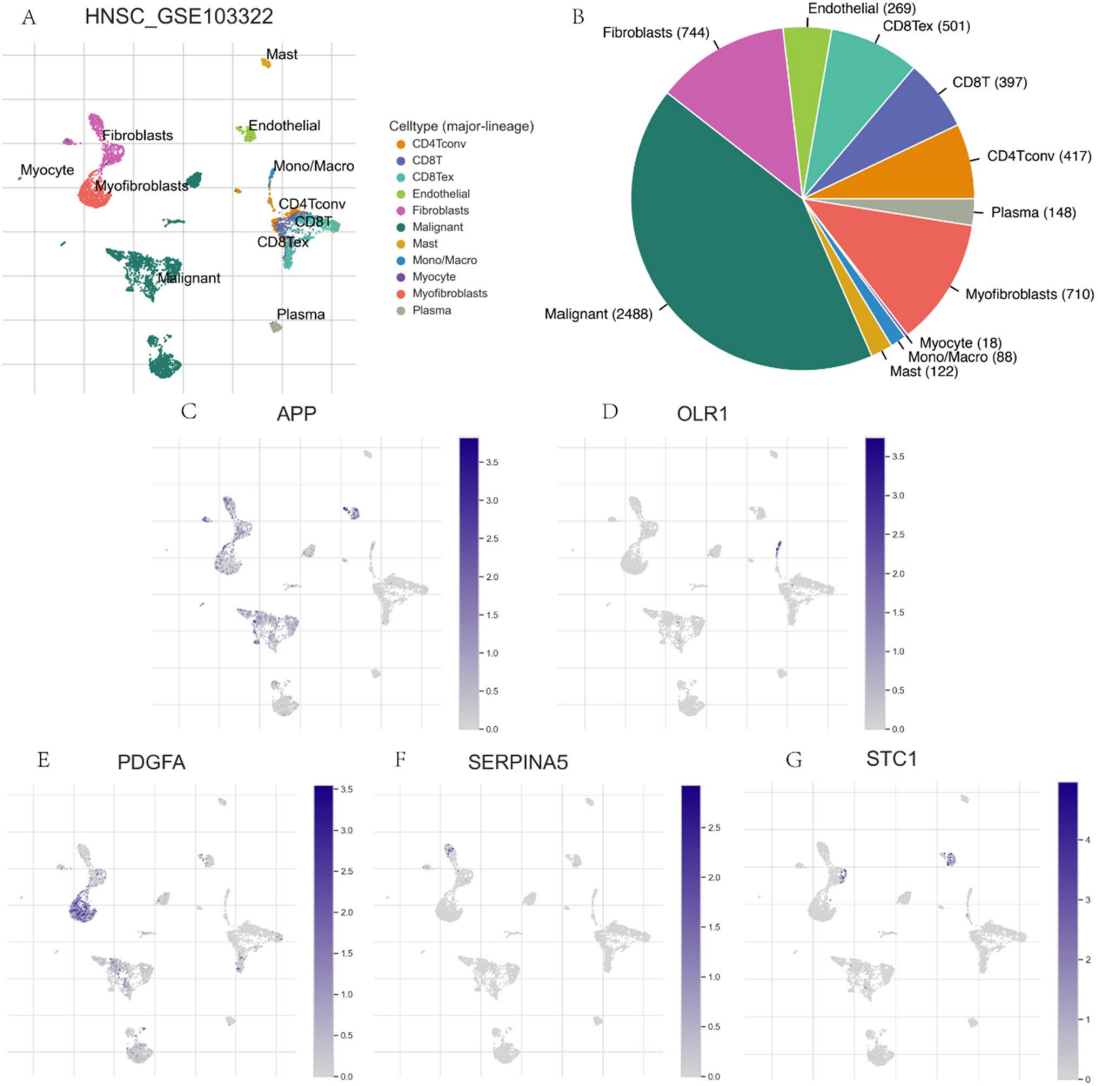
**A**. Cell clustering annotations of UMAP plot in GSE103322 data set. **B**. The number of different cell types in the GSE103322 dataset is shown in a pie chart. The single cell expression of APP **C**, OLR1 **D**, PDGFA **E**, SERPINA5 **F,** and STC1 **G** in GSE103322 cohort (This is the HNSC dataset from the TISCH2 database)

We evaluated the expression of the characterized genes (APP, PDGFA, STC1, OLR1, and SERPINA5) in the HNSC dataset GSE103322 by single cell sequencing using the TISCH2 database. The distribution and number of various cell types in HNSC were presented. The findings demonstrate heightened expression of APP and STC1 in endothelial cells, OLR1 in mono/macro cells, SERPINA5 in fibroblasts, and PDGFA in myofibroblasts (Fig. 8).

## Discussion

In the present investigation, 5 separate genes-namely APP, PDGFA, OLR1, STC1, and SERPINA5 were identified for the creation of a prognostic system. Through the associated survival analysis, it was revealed that those in the high-risk group had substantially shorter OS in comparison to their low-risk group counterparts. The prognostic model of AAGs could function as an independent prognostic determinant for projecting HNSC. More importantly, the five characterized genes were associated with immune infiltration of HNSC, and there was a significant difference in the degree of immune cell infiltration between the high and low risk groups. Additionally, our study identified genes that are associated with metastasis, invasion, and angiogenesis in HNSC.

In recent years, HNSC patients have observed better surgical results when combining radiotherapy, immunotherapy, and targeted therapy simultaneously. These outcomes demonstrate positive advancements in the treatment of HNSC. However, HNSC is prone to local recurrence and metastasis, resulting in an unfavorable prognosis and limited survival [25]. One of the primary factors contributing to the negative forecast for HNSC is linked to neoangiogenesis, which initiates tumor infiltration and consequent metastasis [26]. Angiogenesis is a complex process with both physiological and pathophysiological components, and a delicate balance between pro-angiogenic and anti-angiogenic factors is required for its proper regulation. Interference with this balance can lead to either excessive angiogenesis or angiogenesis inhibition [6]. To date, the role of AAGs in HNSC remains unclear. Thus, in order to enhance the survival and prognosis of HNSC patients, this study aimed to generate a risk-prognostic model of AAGs in HNSC and assess the prognostic significance of the identified genes.

Currently, there are numerous HNSC prognostic models, including those for iron, cellular focal, and copper death. Evidence has emerged to indicate that prognostic models produced by AAGs could be advantageous in evaluating the prognosis of patients with tumors such as gastric adenocarcinoma [27], cervical cancer [28], and glioma [29]. However, additional research is necessary to examine the efficiency of AAGs in the HNSC context. The study screened five AAGs that were prognostically significant in order to build a prognostic model. The model proficiently classifies HNSC patients into high-risk and low-risk cohorts, where the high-risk category entails notably lower OS rates when compared to the low-risk group. The model can act as an autonomous prognostic factor for projecting HNSC. Therefore, these findings confirm that the prognostic model of AAGs possesses good prognostic value, and provides a distinct approach to predicting the prognosis of patients suffering from HNSC.

The invasion of immune cells significantly affects the cure rate and prognosis of HNSC patients [30]. The ssGSEA and EPIC algorithm analysis has shown a correlation between significant immune cell infiltration and HNSC diagnosis in the high-risk group. Furthermore, the research analysis indicates a potential link between immune cell infiltration and tumor angiogenesis. Immunotherapy has proven to be effective in prolonging the lifespan of HNSC patients. Immunotherapy is currently employed as a primary approach for managing metastatic and recurrent HNSC [31]. The analysis revealed significant differences in CD8-T cells and B cells between high and low risk groups. CD8 + T cells have anti-tumor effects, and high levels of CD8 + T cell infiltration correspond to a better prognosis in a variety of cancers [32]. In addition, several studies have shown that B cells have antitumor activity in HNSC [33]. The analysis above showed notable variations in immune cell infiltration between the high- and low-risk groups. Therefore, our study indicates that angiogenesis could be linked to these immune cell infiltrations.

The results of the functional analysis showed that the characterized genes APP, PDGFA, OLR1, STC1, and SERPINA5 were positively correlated with metastasis, invasion, and angiogenesis in HNSC. Similarly, the characterization genes were positively associated with CD34, FN1, LOXL2, and VCAN in HNSC tissues. Additionally, CD34, FN1, LOXL2, and VCAN are key players in tumor angiogenesis, invasion, and metastasis. In particular, CD34 is involved in vascular endothelial cell migration and adhesion [34]. CD34 expression has been reported to increase in HNSC, especially in patients with positive lymph nodes. A significant increase in CD34 cell concentrations was noted [35]. Fibronectin appears to be crucial in cancer pathogenesis and may boost cell proliferation, promote tumor invasion, metastasis, and facilitate angiogenesis [36]. FN1 was significantly upregulated in nasopharyngeal carcinoma tissues, leading to the promotion of cancer cell proliferation and invasion [37]. VCAN can also affect cell adhesion, proliferation, migration, and angiogenesis [38]. Besides, the high VCAN expression in different malignant tumors has been linked to an unfavorable prognosis for patients [39]. LOXL2 showed significant correlation with tumor invasion and progression, and its overexpression may negatively impact the prognosis of HNSC patients [40].

Furthermore, we examined five signature genes. Research has demonstrated that overexpression or ectopic expression of OLR1 can stimulate cancer cell proliferation and expedite the onset, progression, and metastasis of cancer [41]. Additionally, OLR1 high expression is closely associated with an unfavorable prognosis for patients; thus, inhibiting the expression properties of OLR1 is a viable target for the treatment of tumors [42]. In HNSC, the expression of OLR1 is higher than in normal and patients with high OLR1 expression have a poor prognosis [43]. STC1 has been associated with disease progression, survival, metastasis, and negative prognosis in cancer patients [44]. Targeting STC1 can improve the efficacy of tumor immunotherapy [45]. And STC1 is significantly overexpressed in laryngeal squamous cell carcinoma [46]. SERPINA5 is a versatile protein that is downregulated in breast, ovarian, and hepatocellular carcinomas [47]. It has been demonstrated that overexpression of SERPINA5 reduces metastasis, invasion, and angiogenesis of tumor cells [48]. However, the relationship between HNSC and PDGFA is still poorly understood. PDGFA is vital in cell survival, proliferation, migration, and differentiation. High levels of PDGFA have been reported in various cancers, where it promotes cancer cell invasion and proliferation [49]. It has been shown to be an independent predictor of poor patient prognosis [50]. Targeting PDGFA may inhibit metastasis and invasion of oral squamous cell carcinoma [51]. Many cancers have high levels of APP expression. Overexpression of this gene has been linked to increased abnormal growth, migration, and invasion of cancer cells [52]. The expression of APP is upregulated in nasopharyngeal carcinoma tissues, and this gene is also involved in the metastasis and invasion of nasopharyngeal carcinoma [53]. The signature genes of APP, PDGFA, OLR1, STC1, and SERPINA5 are significantly correlated with angiogenesis in HNSC. Further exploration of the relationship between the signature genes and HNSC is necessary when considering AAGs. Anti-angiogenesis treatment provides a promising therapeutic target for HNSC.

In addition, given the significance of chemotherapy as a treatment option for HNSC, we assessed the efficacy of the AAGs model in distinguishing between the outcomes of chemotherapy treatments. According to the findings, patients in the low-risk category exhibited a higher sensitivity to 5-Fluorouracil, Cisplatin, and Paclitaxel in comparison to patients in the high-risk category. Consequently, these results could be a valuable contribution to the personalization of therapy for patients with HNSC.

Nevertheless, it should be noted that our study has certain limitations. Firstly, the study is retrospective, and although relevant data was obtained from public databases for analysis, its heterogeneity may introduce bias. Secondly, the data is assessed solely through bioinformatics analysis. In the future, it will be necessary to advance cellular, histological, in vivo, ex vivo, and clinical trial methods for verifying our conclusions.

## Conclusion

In conclusion, our research in HNSC has identified five critical AAGs associated with patient progression. These genes have important functions in the TME. The constructed prognostic model utilized five genes, and its predictability was subsequently verified. The conclusion drawn is that this predictive model can foresee the prognosis of HNSC patients and aid in directing subsequent treatment. Furthermore, anti-angiogenesis appears as a promising therapeutic target for HNSC. As a result, the aforementioned genes necessitate further scrutiny. This study provides a significant resource for future clinical investigations and explores the relationship between AAGs and HNSC.

## Data Availability

The relevant data used in this study were obtained from the GEO and TCGA databases.
